# Utilization of Artificial Intelligence to support administrative decision-making in special education institutions in Saudi Arabia: perceptions of principals, supervisors, and teachers

**DOI:** 10.3389/frai.2026.1825189

**Published:** 2026-08-03

**Authors:** Abdulaziz Alsuhaymi, Mahmoud Mohamed Eltantawy

**Affiliations:** 1 Department of Educational Management, College of Education, Imam Mohammad Ibn Saud Islamic University (IMSIU), Riyadh, Saudi Arabia; 2 Department of Special Education, College of Education, Imam Mohammad Ibn Saud Islamic University (IMSIU), Riyadh, Saudi Arabia

**Keywords:** administrative decision-making, Artificial Intelligence, perceptions, principals, Saudi Arabia, special education institutions, supervisors, teachers

## Abstract

Artificial Intelligence (AI) is increasingly used in educational administration to automate routine tasks, support data-informed decision-making, and improve institutional efficiency. This study examined the perceptions of principals, supervisors, and teachers regarding the utilization of AI to support administrative decision-making in special education institutions and investigated whether these perceptions differed according to professional role, years of experience, and AI-related training. The sample comprised 173 participants working in public schools providing special education services in Riyadh, Saudi Arabia. A descriptive cross-sectional design was employed, and participants were recruited using non-probability convenience sampling. Data were collected using a 23-item questionnaire and analyzed using descriptive statistics and non-parametric tests. The overall questionnaire score reflected a moderate level of perceptions toward AI utilization (M = 3.22). Participants reported high agreement regarding the importance and usefulness of AI in administrative processes (M = 3.96). In contrast, the ethical considerations dimension yielded a low score (M = 2.38), indicating limited perceived ethical, legal, and professional acceptability of AI utilization and notable reservations regarding the safeguards and conditions governing its responsible use. Perceptions of AI-supported administrative decision-making were moderate (M = 3.07), suggesting that AI was viewed primarily as a supportive resource rather than as a substitute for professional judgment. No statistically significant differences were found according to professional role, *H*(2) = 4.286, *p* = 0.117, or years of experience, *H*(2) = 2.249, *p* = 0.325. Significant differences were found according to AI-related training, *H*(2) = 9.282, *p* = 0.010. Holm-adjusted pairwise comparisons indicated that participants who had completed three to five courses and those who had completed more than five courses obtained significantly higher overall scores than those who had completed fewer than three courses, whereas no statistically significant difference was found between the two higher-training groups. The findings highlight the importance of targeted professional development, ethical safeguards, regulatory clarity, and continued human oversight in the responsible use of AI in special education administration.

## 1 Introduction

Artificial Intelligence (AI) has become increasingly relevant to educational administration because of its capacity to automate routine tasks, analyze large-scale educational data, and support data-informed administrative decision-making. In educational institutions, AI-based systems can assist administrators in planning, monitoring, scheduling, managing records, allocating resources, and generating insights that may improve the efficiency and quality of administrative processes (Ahmad et al., 2022; Jamaica and Tagbo, 2025; Khairullah et al., 2025; Young et al., 2022). Within this context, AI is closely connected to Data-Informed Decision Making (DIDM), which emphasizes the use of educational data to guide decisions and improve institutional performance. However, the use of data-driven systems in education also requires careful attention to ethical, human, and contextual dimensions, particularly when decisions affect vulnerable groups (Daniel, 2015; Huang et al., 2021; Wang, 2019, 2021).

In school administration, AI can support principals and educational staff by streamlining administrative tasks, facilitating communication among stakeholders, reducing workload, and enabling more responsive and evidence-based decision-making (Adams and Thompson, 2025; Ahmad et al., 2022; Sharwani et al., 2025). Rather than replacing educational leaders, AI is increasingly conceptualized as part of a hybrid or symbiotic model in which AI systems perform informational and analytical roles, while human leaders retain responsibility for professional judgment, ethical interpretation, stakeholder negotiation, and final decision-making (Chin, 2025; Dai et al., 2025; Ghamrawi et al., 2024; Zogopoulos and Karatzas, 2024). This distinction is particularly important in special education institutions, where administrative decisions are closely tied to students’ diverse needs, equitable access to services, individualized support, and responsible resource allocation.

Within special education, AI offers promising opportunities to enhance accessibility, inclusion, assistive support, personalized learning pathways, and institutional responsiveness for students with disabilities (Garg and Sharma, 2020; Wani et al., 2025). At the same time, the use of AI in disability-related contexts raises serious ethical concerns, including algorithmic bias, privacy, transparency, accountability, fairness, and the underrepresentation of some disability groups in datasets (Dumitru et al., 2026; Fullan et al., 2024; Mladenov, 2025; Paglialunga and Melogno, 2025; Wang, 2021). These concerns suggest that AI implementation in special education administration should not be examined only as a technical issue, but also as a professional, ethical, and organizational matter that depends on users’ perceptions, competencies, and readiness.

### 1.1 Technology acceptance of AI in educational administration

The present study is theoretically informed by the Technology Acceptance Model (TAM), particularly its concept of perceived usefulness, which provides a relevant lens for interpreting how educational professionals evaluate the potential value of AI in administrative work. TAM is widely used to explain users’ acceptance and use of information technologies (Davis, 1989; Venkatesh and Davis, 2000). Although TAM identifies perceived usefulness and perceived ease of use as two core determinants of technology acceptance, the present study did not operationalize or empirically test the full TAM structure. In particular, perceived ease of use was not represented as a distinct questionnaire dimension. Instead, the study focused on the perceived administrative usefulness of AI, the perceived ethical, legal, and professional acceptability of its utilization, and perceptions of its role in administrative decision-making. Accordingly, TAM was employed as a partial interpretive lens rather than as a complete causal model subjected to empirical testing.

In educational research, TAM has been widely applied to explain teachers’ and students’ acceptance of digital technologies, including learning management systems, educational technology tools, assistive technologies, and AI-based applications (Akbarini, 2024; Ninghardjanti et al., 2026; Runge et al., 2025; Siyam, 2019; Xia et al., 2023). Evidence from these studies indicates that educational technologies are more likely to be accepted when users perceive them as useful for improving performance and sufficiently easy to use in authentic educational contexts. Conversely, acceptance may be reduced when technologies are perceived as complex, time-consuming, insufficiently supported, or weakly aligned with users’ professional tasks and institutional conditions (Siyam, 2019; Xia et al., 2023). In the context of AI, recent research further suggests that AI-related training, AI-related knowledge, and perceived usefulness are important factors shaping educators’ acceptance and use of AI tools (Runge et al., 2025).

The relevance of TAM to the present study lies in its capacity to explain how principals, supervisors, and teachers evaluate AI as a tool for supporting administrative work in special education institutions (Davis, 1989; Venkatesh and Davis, 2000). In this study, AI is not examined as a general technological innovation, but as a decision-support resource that may assist educational professionals in planning, monitoring, resource allocation, task automation, data analysis, and administrative decision-making. Accordingly, perceived usefulness is reflected in participants’ beliefs that AI can improve the quality, efficiency, and data-informed nature of administrative decisions. This includes the perceived capacity of AI to provide timely insights, support predictive analysis, reduce routine administrative workload, enhance knowledge management, and optimize the allocation of educational resources and services. Perceived ease of use is reflected in whether participants perceive AI systems as understandable, accessible, and manageable within their daily professional workload without imposing excessive technical complexity.

For the purposes of the present study, the core constructs were operationally defined as follows. AI utilization refers to participants’ perceptions of the potential and appropriate use of AI-based tools and systems to support administrative functions in special education institutions, including data analysis, planning, monitoring, resource allocation, task automation, and decision support. Perceptions refer to participants’ self-reported evaluations of the administrative usefulness, ethical, legal, and professional acceptability, and decision-support potential of AI in administrative work. Administrative decision-making refers to the process through which school leaders and educational staff use data, professional judgment, institutional values, and regulatory considerations to select appropriate administrative actions related to planning, service delivery, resource distribution, and support for students with disabilities.

Because the present study is descriptive and perception-based, TAM is used as a theoretical lens rather than as a full causal model to be tested statistically. The questionnaire dimensions were therefore interpreted in relation to TAM and the specific context of AI-supported administration in special education. The first dimension, “the importance of AI in the administrative process,” reflects perceived usefulness because it captures participants’ views of AI as a tool for improving administrative efficiency, data-informed planning, and institutional performance. The second dimension, “ethical considerations of AI utilization,” captures contextual and ethical factors that may shape technology acceptance, particularly in special education settings where decisions involve students’ rights, equity, privacy, fairness, and access to appropriate services. The third dimension, “AI and administrative decision-making,” reflects participants’ views of the role, limits, and acceptability of AI-supported decisions within educational institutions.

This theoretical framework is especially appropriate for special education administration because acceptance of AI in this field cannot be understood solely as a technical matter. Prior research indicates that technology acceptance among special education teachers may be influenced by access to technology, time, self-efficacy, job relevance, training, and support conditions (Siyam, 2019). Similarly, research on AI acceptance suggests that AI-related training and competence can influence users’ perceptions of AI usefulness and use (Runge et al., 2025). At the same time, the responsible use of AI in disability-related contexts is complicated by concerns about data bias, the underrepresentation of some disability groups in datasets, and the use of non-representative data (Dumitru et al., 2026; Mladenov, 2025; Paglialunga and Melogno, 2025; Wang, 2021). These concerns indicate that AI acceptance in special education institutions should be examined through technical, professional, contextual, and ethical dimensions.

Despite the growing use of TAM in educational research, important gaps remain in the literature on artificial intelligence in special education administration. Existing studies have largely examined teachers’ or students’ acceptance of educational technologies and, more recently, AI-based applications in teaching and learning contexts, with limited attention to the administrative use of AI in schools and educational institutions (Akbarini, 2024; Runge et al., 2025; Xia et al., 2023). In particular, current literature provides insufficient evidence on how professional role, years of experience, and AI-related training may shape educators’ perceptions of AI when it is used to support administrative decision-making rather than classroom instruction.

This gap is especially salient in special education settings, where administrative decisions are tightly linked to students’ diverse needs, equitable service provision, and responsible resource allocation. While prior studies grounded in TAM have examined teachers’ and students’ acceptance of educational technologies and AI applications primarily in teaching and learning contexts, relatively little is known about how principals, supervisors, and teachers in special education institutions perceive AI when it is used specifically to support administrative decision-making (Siyam, 2019; Xia et al., 2023; Runge et al., 2025; Ninghardjanti et al., 2026). Moreover, limited evidence is available on whether these perceptions differ according to professional role, years of experience, and AI-related training. These variables are important because principals, supervisors, and teachers may encounter AI through different administrative responsibilities, levels of decision-making authority, and opportunities for professional development.

Against this background, the present study aims to investigate the perceptions of principals, supervisors, and teachers regarding the utilization of AI in supporting administrative decision-making within special education institutions in Saudi Arabia, and to examine whether these perceptions differ significantly by professional role, years of experience, and prior AI-related training. The Saudi context is particularly relevant because the integration of AI into educational administration aligns with the national direction toward digital transformation, efficiency, and innovation under Saudi Vision 2030 (Vision 2030, 2016) and is supported by the ethical and regulatory frameworks advanced by the Saudi Data and Artificial Intelligence Authority (2022). Clarifying these perceptions can provide empirical evidence to guide the responsible and sustainable implementation of AI in special education administration.

Accordingly, the present study addresses the following questions:

- What are the perceptions of principals, supervisors, and teachers regarding the utilization of AI in administrative decision-making within special education institutions?
- Are there statistically significant differences in these perceptions attributable to professional role, years of experience, or AI-related training?

## 2 Method

### 2.1 Study design

To achieve the objectives of this study, a descriptive cross-sectional design was employed, using a questionnaire as the primary data collection tool. Descriptive research is widely recognized as one of the most appropriate methodologies for describing educational phenomena as they exist in practice and for investigating attitudes and perspectives of specific groups regarding a particular issue (Best and Kahn, 2016). This design was effectively applied in the current study by investigating perceptions of principals, supervisors, and teachers.

### 2.2 Participants and sampling frame

The study sample comprised 173 participants, including principals, supervisors, and teachers working in public schools in Riyadh that provide special education services. Participants were selected using a non-probability convenience sampling approach. The sampling frame included eligible professionals working in public special education schools or special education programs attached to public schools during the 2025–2026 academic year. Access to participants was facilitated through official authorization and coordination with the Saudi Ministry of Education. Data were collected over a two-month period, from October to November 2025, which is important to specify given the rapidly evolving role of AI in educational administration.

The target population consisted of principals, supervisors, and teachers directly involved in special education administration or service provision within this institutional and geographical context. Because the exact number of eligible professionals was not available to the researchers through an accessible official dataset, the population was delimited by professional role, institutional setting, and location rather than by a fixed numerical size.

To evaluate the adequacy of the obtained sample, an *a priori* power analysis was conducted using G*Power, a widely used program for statistical power analysis in the behavioral and social sciences (Faul et al., 2007). Because the main inferential analyses involved comparisons across three independent groups, the calculation was based on a fixed-effects omnibus one-way ANOVA approximation. This approach was used to estimate the required sample size for group-comparison analyses, including non-parametric alternatives such as the Kruskal–Wallis test. The analysis was based on Cohen’s effect size index for one-way group comparisons, calculated as *f* = √[*η*^2^/(1 − *η*^2^)], where *η*^2^ represents the proportion of variance explained by group differences. Following Cohen’s conventions, a medium effect size was set at *f* = 0.25 (Cohen, 2013). The following parameters were entered into G*Power: effect size *f* = 0.25, alpha level = 0.05, desired statistical power = 0.80, and number of groups = 3. Based on these parameters, the minimum required sample size was approximately 158 participants. Therefore, the final sample of 173 exceeded this requirement and provided an estimated statistical power of approximately 0.84.

In this study, “administrators” referred to school principals and administrative leaders directly responsible for managing special education programs or services, rather than general clerical or support staff. “Supervisors” referred to educational supervisors and special education program coordinators responsible for monitoring program implementation, supporting staff, and overseeing the delivery of special education services at the school or education-department level. “Teachers” referred to teachers working directly in special education schools or programs and involved in the educational and administrative processes associated with the provision of services to students with disabilities.

Participants were included if they: (a) were currently employed as administrators, supervisors, or teachers in eligible public schools providing special education services; (b) had direct involvement in administrative and/or pedagogical processes related to special education, such as planning, monitoring, resource allocation, program coordination, or service provision; and (c) provided informed consent. Individuals were excluded if they were administrative, clerical, or support staff with no direct responsibilities for special education, teachers working exclusively in general education classrooms, or individuals who did not consent to participate. No submitted questionnaires were excluded because all survey items were designated as mandatory on the electronic platform before submission.

Several potential sources of bias were considered. First, because the study was limited to public schools in Riyadh, the sample may reflect geographic and urban bias, as the city has comparatively stronger digital infrastructure, connectivity, administrative resources, and access to professional development opportunities than some rural or less-resourced regions. Second, socioeconomic and infrastructural bias may be present because participants’ perceptions of AI could be influenced by digital access, institutional support, technological readiness, and resource availability in their schools. Third, the use of non-probability convenience sampling may limit the representativeness of the findings. Fourth, reliance on self-reported questionnaire data may introduce response bias and social desirability bias.

To reduce these potential biases, the study boundaries were clearly defined, and the findings were interpreted within the specific context of public special education settings in Riyadh rather than generalized to all special education institutions in Saudi Arabia. Data collection procedures were standardized through clear instructions and consistent administration procedures. The electronic questionnaire link was distributed to eligible participants through school principals and/or program coordinators. Participation was entirely voluntary, and respondents were assured of confidentiality and anonymity. To further reduce potential social desirability bias, there was no direct interaction with the researchers during the response process.

Table 1 presents the demographic characteristics of the participants, including their distribution by gender, professional role, educational stage, AI-related training, and years of experience.

**Table 1:** Demographic characteristics of study participants.

Variable	Category	*N*	%
Gender	Male	112	64.7
	Female	61	35.3
	Total	173	100.0
Professional role	Administrators	23	13.3
	Supervisors	61	35.3
	Teachers	89	51.4
	Total	173	100.0
Educational stage	Elementary stage	69	39.9
	Middle school	63	36.4
	High school	41	23.7
	Total	173	100.0
AI-related training	Fewer than 3 courses	57	32.9
	From 3 to 5 courses	56	32.4
	More than 5 courses	60	34.7
	Total	173	100.0
Years of experience	From 5 years to less than 10 years	55	31.8
	From 10 years to less than 15 years	66	38.2
	More than 15 years	52	30.0
	Total	173	100.0

### 2.3 Tools

To achieve the study objectives and address the research questions, a questionnaire titled “Perceptions of principals, supervisors, and teachers toward the utilization of Artificial Intelligence in administrative decision-making” was developed. The questionnaire was designed to assess participants’ perceptions regarding integration of AI into administrative decision-making processes. The tool was developed based on a comprehensive review of relevant literature, including research by Best and Kahn (2016) and Arrooqi and Alruqi (2025).

The initial version of the questionnaire comprised 26 items covering several content areas, including participants’ perceptions of AI-powered administration, data literacy and analytics skills, the role of AI in streamlining administrative tasks in the educational process, resource planning and optimization, ethical and legal considerations of AI, psychosocial implications, and the perceived role of AI in supporting administrative decision-making. Responses were recorded using a five-point Likert-type scale, with scores ranging from 1 to 5.

#### 2.3.1 Content validity

Content validity of the questionnaire was examined through expert review, in alignment with practices commonly used in instrument development (Mengual-Andrés et al., 2016). The validation process involved four referees from Saudi academic institutions, including two specialists in special education and two specialists in educational administration. The experts reviewed the initial version of the questionnaire to evaluate the conceptual relevance of the items, the clarity of wording, the appropriateness of the items for the Saudi special education context, and the alignment of each item with its proposed dimension. They were also asked to identify redundant, overlapping, or conceptually ambiguous items that could contribute to construct contamination across questionnaire domains.

Based on the experts’ feedback, two items were removed, several items and concepts were simplified, and linguistic adjustments were made to enhance clarity and reduce overlap between domains. Consequently, the revised version of the questionnaire comprised 24 items. Because the expert panel consisted of four reviewers, the content validation process was treated as qualitative expert-based evidence rather than as a comprehensive quantitative content validity assessment.

#### 2.3.2 Pilot testing

Following the content validation stage, a pilot test was conducted with 14 principals, supervisors, and teachers working in special education programs. The purpose of the pilot test was not to conduct psychometric validation or factor analysis, but to assess participants’ comprehension of the questionnaire items, the clarity and precision of the phrasing, the readability of the instructions, and the feasibility of completing the electronic questionnaire. Feedback from the pilot stage resulted in the refinement and simplification of several items to improve readability, while one additional item was removed. Consequently, the final version of the questionnaire comprised 23 items. It should be noted that the pilot sample was used only for clarity and feasibility checking and was not used for Exploratory Factor Analysis. The Exploratory Factor Analysis and reliability analysis were conducted using the main study sample of 173 participants.

#### 2.3.3 Exploratory Factor Analysis

To examine preliminary evidence of construct validity for the final 23-item questionnaire, Exploratory Factor Analysis (EFA) was conducted on the main study sample of 173 participants using Principal Axis Factoring (PAF). This analysis was intended to examine the questionnaire’s dimensional structure and determine whether the items clustered empirically in a manner consistent with the theoretically proposed domains. The results of the Kaiser–Meyer–Olkin (KMO) measure of sampling adequacy yielded a high value of 0.818, exceeding the minimum acceptable threshold of 0.60 for factor analysis as recommended by Kaiser (1974). Additionally, Bartlett’s Test of Sphericity reached statistical significance at *p* < 0.001, confirming that the correlation matrix was suitable for factor analysis.

The analysis extracted three factors with eigenvalues greater than 1, accounting for 49.499% of the total variance. The first factor, comprising 10 items, was titled “The importance of AI in the administrative process”; the second factor, consisting of 8 items, was titled “ethical considerations of AI utilization”; and the third factor, containing 5 items, was titled “AI and administrative decision-making.” Overall, the extracted factor structure provided preliminary support for the three-domain model by showing that the items clustered into three interpretable factors that were conceptually aligned with the theoretical framework of the study. Nevertheless, the possibility of partial construct overlap among domains cannot be fully ruled out and is acknowledged as a limitation to be addressed through further refinement and Confirmatory Factor Analysis (CFA) in future research. These results are detailed in Table 2.

**Table 2:** Summary of Exploratory Factor Analysis.

Items	Factor 1	Factor 2	Factor 3
Q 7	0.780		
Q 8	0.767		
Q 9	0.755		
Q 11	0.748		
Q 6	0.745		
Q 3	0.704		
Q 10	0.690		
Q 1	0.688		
Q 2	0.665		
Q 4	0.612		
Q 13		0.741	
Q 15		0.721	
Q 12		0.700	
Q 14		0.609	
Q 19		0.590	
Q 20		0.554	
Q 16		0.545	
Q 21		0.513	
Q 17			0.748
Q 22			0.732
Q 18			0.679
Q 5			0.509
Q 23			0.461

Based on the EFA results and the theoretical framework of the study, the final questionnaire was operationalized into three dimensions. The first dimension, “The importance of AI in the administrative process,” included 10 items assessing participants’ perceptions of the usefulness of AI in improving administrative efficiency, supporting data-informed planning, enhancing knowledge management, streamlining routine administrative tasks, and optimizing resource allocation within special education institutions. The second dimension, “ethical considerations of AI utilization,” included 8 items assessing participants’ evaluations and concerns regarding the ethical, legal, and professional implications of AI use in administrative processes, including fairness, accountability, privacy, compliance with regulations, human values, and the potential effects of AI on professional relationships. The third dimension, “AI and administrative decision-making,” included 5 items assessing participants’ perceptions of the role, limits, and acceptability of AI-supported administrative decisions, including issues of decision quality, accountability, fairness, time requirements, and the possibility of AI replacing or constraining human leadership.

All items were rated on a five-point Likert-type scale ranging from 1 to 5 and were positively worded and scored in the same direction. Higher scores therefore indicated stronger agreement with the item statements. Dimension scores were calculated by averaging the responses to the items within each dimension, whereas the total questionnaire score was calculated by averaging the responses to all 23 items. Higher scores on the first dimension indicated more favorable perceptions of the importance and usefulness of AI in administrative processes. Higher scores on the second dimension indicated greater perceived ethical, legal, and professional acceptability of AI utilization, as well as greater confidence in the conditions and safeguards governing its responsible use. Higher scores on the third dimension reflected more favorable perceptions of AI-supported administrative decision-making. Accordingly, higher total questionnaire scores indicated more favorable overall perceptions of AI utilization in administrative decision-making.

#### 2.3.4 Questionnaire reliability

The reliability of the questionnaire was assessed using Cronbach’s alpha coefficient. The results yielded high reliability scores across all dimensions: the first dimension attained an alpha of 0.900, the second dimension 0.775, and the third dimension 0.703. The overall reliability was 0.787.

### 2.4 Data collection and statistical analysis

After the questionnaire had been finalized, ethical approval was obtained from the Institutional Review Board (IRB) at Imam Mohammad Ibn Saud Islamic University, followed by official authorization from the Saudi Ministry of Education. The Ministry facilitated questionnaire administration by distributing the electronic survey link to school principals, who subsequently forwarded it to the target participants. The questionnaire included an introductory section providing general information about the study, its purpose, and participation procedures. Participants were informed that their participation was voluntary, that their responses would remain confidential, and that they had the right to withdraw at any time. Informed consent was required before completing the questionnaire.

To address the research questions and examine the psychometric properties of the questionnaire, several statistical procedures were employed. Descriptive statistics were calculated for the questionnaire items, and dimension and total scores were computed by averaging the relevant item responses. Higher mean scores indicated stronger agreement with the relevant item or dimension content.

The Kolmogorov–Smirnov test was used to assess the normality of the data distribution. The results indicated statistically significant deviations from normality across the study variables (*p* < 0.05). Levene’s test was conducted to examine the homogeneity of variances, and the results indicated that the assumption of homogeneity was met (*p* > 0.05). Given the non-normal distribution of the data, the Kruskal–Wallis test was employed to examine statistically significant differences in participants’ perceptions according to professional role, years of experience, and AI-related training.

When the Kruskal–Wallis test yielded a statistically significant result, pairwise Mann–Whitney *U* tests were conducted as non-parametric post-hoc comparisons to identify the sources and direction of differences between groups. To control the familywise Type I error rate across the multiple pairwise comparisons, the Holm–Bonferroni sequential correction was applied. Statistical significance was determined using Holm-adjusted *p*-values at an overall alpha level of 0.05. Cronbach’s alpha coefficient was used to assess the internal consistency reliability of the questionnaire and its dimensions. All analyses were conducted using IBM SPSS Statistics, Version 27.

## 3 Results

### 3.1 Demographic characteristics of the participants

The study sample consisted of 173 participants working in special education settings. The majority were male (64.7%), while females represented 35.3% of the sample. Most participants were teachers (51.4%), followed by supervisors (35.3%) and administrators (13.3%). In terms of educational stage, 39.9% worked in elementary schools, 36.4% in middle schools, and 23.7% in high schools. Regarding AI-related training, 32.9% of participants had completed fewer than three training courses, 32.4% had completed three to five courses, and 34.7% had completed more than five courses. With respect to years of experience, 31.8% had from 5 to less than 10 years of experience, 38.2% had from 10 to less than 15 years, and 30.0% had more than 15 years of experience.

After describing the demographic characteristics, the following subsections present the major findings regarding participants’ perceptions of AI utilization in administrative decision-making and the significant group differences.

### 3.2 Descriptive statistics of the questionnaire scores

To address the first research question concerning participants’ perceptions of AI utilization in administrative decision-making, item-level means and standard deviations were calculated for the questionnaire. Participants’ responses were interpreted according to the following criteria: very low level (1.00–1.79), low level (1.80–2.59), moderate level (2.60–3.39), high level (3.40–4.19), and very high level (4.20–5.00). The results are presented in Table 3.

**Table 3:** Means and standard deviations of participants’ perceptions.

No.	Item	M	SD
Dimension 1: significance of AI in administrative processes			
7	The utilization of technological tools within educational institutions facilitates accurate administrative decision-making.	4.03	0.702
8	The adoption of modern management systems by the educational institution (e.g., AI-powered administration) enhances administrative decision-making processes.	3.97	0.792
9	Data literacy and analytical skills are essential components of administrative decision-making within educational institutions.	3.96	0.693
11	A human element proficient in utilizing AI tools is a vital element in administrative decision-making.	4.05	0.693
6	AI facilitates data-driven administrative decision-making.	3.99	0.682
3	Artificial Intelligence plays a significant role in streamlining and simplifying administrative tasks within the educational process.	4.12	0.730
10	AI plays a pivotal role in the automation of administrative processes.	3.87	0.744
1	AI significantly contributes to resource planning and optimization (e.g., proposing enhanced strategies for school resource allocation).	3.85	0.716
2	AI supports predictive analytics within educational institutions.	3.65	0.782
4	The utilization of AI has a positive impact on knowledge management.	4.11	0.838
	*Overall dimension score*	3.96	0.54
Dimension 2: ethical considerations of AI utilization			
13	AI is used in administrative decision-making within educational institutions in a manner that upholds core ethical principles and values.	2.27	0.769
15	AI contributes to generating outputs that are consistent with the practical and real-world conditions of the educational institution.	2.51	0.846
12	AI can be used efficiently in administrative processes when appropriate requirements and safeguards are in place.	2.35	0.775
14	Regulatory and legal safeguards can help ensure that AI-supported administrative decisions comply with applicable laws and regulations.	2.53	0.796
19	AI is an appropriate tool for supporting administrative decision-making, while final decisions remain under human professional responsibility.	2.49	0.893
20	AI can be used in a manner that preserves professional relationships, human interaction, and empathy within administrative teams.	2.24	0.799
16	AI helps reduce the influence of human emotions and personal sentiments on administrative decisions.	2.40	0.981
21	AI helps reduce the influence of social pressures, such as peer influence in the workplace, on administrative decisions.	2.27	0.746
	*Overall dimension score*	2.38	0.52
Dimension 3: AI and administrative decision-making			
17	I believe that AI has the potential to support school leaders and enhance their effectiveness in the future.	3.29	0.747
22	Decision-makers can maintain accountability for decisions reached through the integration of AI technologies.	2.54	1.128
18	The implementation of AI in administrative decision-making can help save time and improve efficiency.	3.46	0.796
5	Administrative decisions involving AI are characterized by fairness and equity.	2.54	1.026
23	Administrative decisions driven by AI may exhibit a high degree of excellence and distinction.	3.50	0.919
	*Overall dimension score*	3.07	0.58
	*Total score for the entire questionnaire*	3.22	0.34

Table 3 indicates that participants perceived the significance of AI in administrative processes as high (M = 3.96, SD = 0.54). The ethical considerations dimension yielded a low mean score (M = 2.38, SD = 0.52), indicating low perceived ethical acceptability of AI utilization in administrative decision-making and suggesting notable ethical and professional reservations. Participants’ perceptions of the role of AI in administrative decision-making were moderate (M = 3.07, SD = 0.58). The overall mean across the 23 questionnaire items was 3.22, indicating a moderate overall evaluation of AI utilization in administrative decision-making.

### 3.3 Group differences in perceptions by demographic variables

To address the second research question, the Kruskal–Wallis test was employed to compare participants’ perceptions according to professional role, AI-related training, and years of experience, due to the non-normal distribution of the data. The results of these analyses are presented in Tables 4–6.

**Table 4:** Kruskal–Wallis test for group comparisons based on professional role.

Variable	Group	*N*	Mean rank	*H*	df	*p*
Overall questionnaire score	Administrators	23	84.02	4.286	2	0.117
	Supervisors (program coordinators, educational supervisors in departments)	61	97.56			
	Teachers	89	80.53			
	Total	173				

**Table 5:** Kruskal–Wallis test for group comparisons based on AI-related training.

Variable	Group	*N*	Mean rank	*H*	df	*p*
Overall questionnaire score	Fewer than 3 courses	57	70.84	9.282	2	0.010
	From 3 to 5 courses	56	91.86			
	More than 5 courses	60	97.82			
	Total	173				

**Table 6:** Kruskal–Wallis test for group comparisons based on years of experience.

Variable	Group	*N*	Mean rank	*H*	df	*p*
Overall questionnaire score	From 5 years to less than 10 years	55	83.73	2.249	2	0.325
	From 10 years to less than 15 years	66	82.89			
	More than 15 years	52	95.68			
	Total	173				

Table 4 shows that the Kruskal–Wallis test was not statistically significant for professional role, *H*(2) = 4.286, *p* = 0.117, indicating that participants’ perceptions did not differ significantly by professional role.

As illustrated in Table 5, the Kruskal–Wallis test revealed a statistically significant difference in the overall questionnaire score according to AI-related training, *H*(2) = 9.282, *p* = 0.010. The mean ranks increased across the training categories, from 70.84 among participants who had completed fewer than three courses to 91.86 among those who had completed three to five courses and 97.82 among those who had completed more than five courses. Pairwise Mann–Whitney *U* tests were subsequently conducted, and the Holm–Bonferroni sequential correction was applied to control the familywise Type I error rate.

The results indicated that participants who had completed three to five courses obtained significantly higher overall questionnaire scores than those who had completed fewer than three courses, *U* = 1181.500, *Z* = −2.384, Holm-adjusted *p* = 0.034. Participants who had completed more than five courses also obtained significantly higher scores than those who had completed fewer than three courses, *U* = 1203.500, *Z* = −2.766, Holm-adjusted *p* = 0.018. However, no statistically significant difference was found between participants who had completed three to five courses and those who had completed more than five courses, *U* = 1537.500, *Z* = −0.789, Holm-adjusted *p* = 0.430. Thus, the overall difference was attributable to the lower scores of participants who had completed fewer than three AI-related training courses compared with both groups that had received more extensive training.

Table 6 shows that the Kruskal–Wallis test was not statistically significant for years of experience, *H*(2) = 2.249, *p* = 0.325, indicating that participants’ perceptions did not differ significantly by years of experience.

## 4 Discussion

The findings for the first research question indicated an overall moderate level of perceptions regarding the utilization of AI in administrative decision-making. However, the results varied across the three questionnaire dimensions, revealing a pattern that combined high perceived administrative usefulness, substantial ethical concern, and moderate acceptance of AI-supported administrative decision-making.

The first dimension, “The importance of AI in the administrative process,” received a high level of agreement, indicating that participants recognized the potential significance of AI in supporting administrative processes. From a Technology Acceptance Model (TAM) perspective, this finding may reflect relatively high perceived usefulness. Participants appeared to regard AI as a potentially valuable resource for supporting data-informed administrative decision-making, providing analytical insights, streamlining routine tasks, improving knowledge management, and assisting with resource planning and allocation. This pattern is consistent with the TAM assumption that technologies are more likely to be viewed favorably when users perceive them as capable of improving job performance and task effectiveness. These findings align with those of several previous studies (Jamaica and Tagbo, 2025; Sharwani et al., 2025; Young et al., 2022). The high perceived importance of AI may also be related to the complex administrative and educational responsibilities associated with special education, where professionals must coordinate services and respond to the diverse educational support needs of students with disabilities. In this regard, Garg and Sharma (2020) and Wani et al. (2025) highlight the potential value of AI for individuals with disabilities and the inclusive educational institutions that serve them.

Regarding the third dimension, “AI and administrative decision-making,” participants reported a moderate level of perceptions. From a TAM perspective, this result may indicate that although participants recognized some usefulness in AI-supported decision-making, they remained cautious about the extent to which AI should be relied upon in sensitive administrative contexts. Participants showed relatively stronger agreement regarding the potential of AI to save time, improve efficiency, enhance decision quality, and support school leaders. However, their responses to items concerning accountability, fairness, and equity indicated continuing reservations about the reliability and acceptability of decisions supported by AI. Thus, participants did not appear to reject the role of AI in administrative decision-making, but neither did they demonstrate unconditional acceptance of autonomous AI-based decisions. These concerns may limit the acceptance of AI even when it is regarded as administratively useful, particularly when professional accountability, human judgment, and contextual interpretation remain essential. These results are consistent with the findings of Ghamrawi et al. (2024), which suggest that AI may challenge traditional leadership roles through increased automation. Similarly, these results align with the perspective of Dai et al. (2025), who advocate for the development of frameworks in which AI augments and enhances educational leadership rather than replacing it.

The second dimension, “ethical considerations of AI utilization,” yielded a low level of agreement. Because all items were positively worded and scored in the same direction, this finding indicates limited perceived ethical, legal, and professional acceptability of AI utilization in administrative decision-making. Participants reported relatively low confidence that AI could be used in ways that uphold ethical principles and human values, generate outputs appropriate to the practical context of educational institutions, comply with applicable laws and regulations, and preserve professional relationships, human interaction, and empathy. They also expressed limited confidence in the suitability of AI as a decision-support tool operating under human professional responsibility, in the adequacy of the safeguards governing its use, and in its capacity to reduce the influence of personal emotions and social pressures on administrative decisions. These findings suggest notable ethical, legal, and professional reservations regarding AI utilization, despite participants’ recognition of its potential administrative benefits.

Although TAM primarily emphasizes perceived usefulness and perceived ease of use, the present findings suggest that ethical and contextual concerns may constitute important surrounding conditions that influence the acceptance of AI in special education administration. In particular, concerns about fairness, privacy, accountability, legal compliance, human values, and the rights of individuals with disabilities may restrict acceptance even when AI is viewed as useful. These findings are consistent with studies emphasizing the importance of integrating ethical and legal safeguards into administrative processes (Karakuş et al., 2025; Khairullah et al., 2025; Madanchian and Taherdoost, 2025; Wang, 2021) and mitigating potential biases against individuals with disabilities (Paglialunga and Melogno, 2025). The Saudi context should also be considered when interpreting this result. Arrooqi and Alruqi (2025) indicated that ethical considerations are among the factors affecting the readiness of academic leadership to implement AI in Saudi Arabia. Moreover, the ethical and regulatory frameworks established by the Saudi Data and Artificial Intelligence Authority (2022) emphasize the need for fairness, accountability, transparency, privacy, and responsible human oversight.

Taken together, the results indicate a cautious and balanced position toward AI utilization in the sampled special education institutions. Participants recognized the administrative usefulness of AI but expressed substantial ethical concern and only moderate acceptance of its direct role in administrative decision-making. From a TAM-related perspective, high perceived usefulness may encourage interest in AI adoption, whereas ethical concerns and reservations about accountability and fairness may restrict unconditional acceptance. The moderate overall level therefore reflects neither rejection nor complete acceptance of AI; rather, it indicates conditional support for its utilization as a complementary administrative resource under continued human supervision and professional accountability.

Concerning the second research question, the results indicated no statistically significant differences in participants’ overall questionnaire scores attributable to professional role or years of experience. This suggests that, within the present sample, principals, supervisors, and teachers, as well as participants with different levels of professional experience, reported relatively similar overall evaluations of the administrative usefulness, ethical implications, and decision-support potential of AI. From a TAM perspective, this pattern may indicate that the perceived benefits and concerns associated with AI were relatively shared across professional roles and experience levels rather than being strongly differentiated by position or tenure. The findings may also reflect a shared recognition of the potential role of AI in supporting educational and administrative processes for students with disabilities. In this context, Garg and Sharma (2020) and Wani et al. (2025) emphasize the potential of AI to support accessibility, inclusion, and empowerment for individuals with disabilities. Additionally, Arrooqi and Alruqi (2025) reported that academic leaders in Saudi Arabia demonstrated confidence in AI and its perceived benefits, which may support its integration and utilization.

With respect to AI-related training, the results revealed statistically significant differences in participants’ overall questionnaire scores. Holm-adjusted pairwise comparisons indicated that participants who had completed three to five courses and those who had completed more than five courses obtained significantly higher scores than participants who had completed fewer than three courses. However, no statistically significant difference was found between the two higher-training groups. Because the questionnaire items were coded in a consistent direction, higher overall scores reflected more favorable evaluations of AI utilization, including higher perceived administrative usefulness, lower ethical concern, and greater openness to the decision-support role of AI.

This pattern may indicate that exposure to a sufficient level of AI-related training is associated with more favorable perceptions, but that increasing the number of courses beyond a certain level does not necessarily result in a corresponding improvement in perceptions. This interpretation is consistent with Kazmaci et al. (2025), who found that theoretical and practical knowledge of AI contributed to teachers’ capacity for sustainable AI integration, partly through its influence on their beliefs and attitudes. Training may therefore improve participants’ understanding of AI applications, clarify their professional relevance, reduce uncertainty about their use, and strengthen confidence in their responsible implementation. The findings also correspond with Qi (2025), who identified inadequate training as an important factor underlying the gap between educators’ generally positive perceptions of AI and their practical readiness to implement it. Participants with fewer than three courses may consequently have had more limited opportunities to develop the knowledge and confidence required to evaluate AI favorably in administrative contexts.

From a TAM perspective, AI-related training may be associated with greater perceived usefulness and familiarity with AI tools. However, the absence of a significant difference between the two higher-training groups suggests that the quality and practical relevance of training may be more important than the number of courses completed. Given the cross-sectional design, these findings indicate association rather than causation. Overall, the limited perceived ethical, legal, and professional acceptability of AI utilization, together with moderate acceptance of AI-supported decision-making, supports the use of AI as a complementary administrative resource under continued human oversight and professional accountability.

However, several limitations of this study must be acknowledged. First, the use of non-probability convenience sampling to recruit participants limits the generalizability of the findings beyond the sampled principals, supervisors, and teachers and may introduce self-selection bias. Second, the validation evidence for the questionnaire should be interpreted as preliminary. Although the instrument development process included expert review, pilot testing, Exploratory Factor Analysis (EFA), and internal consistency reliability analysis, the content validation stage relied on a limited panel of four experts, and the pilot sample was used only to assess item clarity, readability, and feasibility rather than to establish full psychometric validation. In addition, although EFA supported the three-factor structure of the questionnaire, Confirmatory Factor Analysis (CFA) was not performed, which restricts the ability to test the stability of the measurement model across different samples. The possibility of partial construct overlap among questionnaire domains also cannot be fully ruled out. Third, the study relied exclusively on descriptive, cross-sectional self-report survey data; therefore, the findings reflect participants’ perceptions rather than their actual use of AI systems or the observed quality of AI-supported administrative decision-making. Subsequent research would benefit from mixed-methods or longitudinal designs to provide deeper and more nuanced insights into how perceptions of AI evolve over time and in relation to actual implementation. Fourth, the categorization of years of experience did not include more fine-grained subgroups, such as novice teachers with fewer than 3 years of experience or highly experienced leaders with more than 20 years, which may have limited the detection of more subtle differences in perceptions across experience levels. Finally, the study was conducted solely in public schools in Riyadh, an urban context characterized by comparatively advanced services, stronger digital infrastructure, and greater access to professional development than many rural or less-resourced regions. This urban, infrastructural, and socioeconomic context should be considered when interpreting the findings, as it limits the extent to which the results can be generalized to all special education institutions in Saudi Arabia. Future studies should include participants from different regions, including urban, suburban, and rural settings, to provide a more representative understanding of AI utilization in special education administration.

## 5 Conclusion

This study examined the perceptions of principals, supervisors, and teachers regarding the utilization of Artificial Intelligence (AI) to support administrative decision-making in public schools providing special education services in Riyadh, Saudi Arabia. The findings indicated that participants recognized the importance and potential usefulness of AI in administrative processes, including data-informed planning, task automation, knowledge management, and resource allocation.

However, the low level of agreement on the ethical considerations dimension indicated limited perceived ethical, legal, and professional acceptability of AI-supported administrative practices. Participants reported relatively low confidence that AI could be used in ways that uphold ethical principles and human values, generate outputs appropriate to the practical context of educational institutions, comply with applicable laws and regulations, preserve professional relationships and empathy, and function within appropriate conditions of human responsibility and institutional safeguards. Perceptions of AI-supported administrative decision-making were moderate, suggesting that participants recognized its potential value as a decision-support resource while remaining cautious about extensive reliance on it. Overall, the findings reflect a balanced position in which recognition of AI’s potential administrative usefulness coexisted with reservations regarding its ethical, legal, and professional acceptability and its direct role in administrative decision-making.

No statistically significant differences were found in total questionnaire scores according to professional role or years of experience. In contrast, statistically significant differences were identified according to AI-related training. Participants who had completed three to five courses and those who had completed more than five courses obtained significantly higher total scores than participants who had completed fewer than three courses, whereas the two higher-training groups did not differ significantly. These findings indicate that greater exposure to AI-related training was associated with more favorable perceptions of AI utilization. However, the cross-sectional design does not permit causal conclusions, and the absence of a significant difference between the two higher-training groups does not establish whether the number, quality, content, or practical relevance of training accounted for this pattern.

In practical terms, educational authorities and schools may consider developing context-specific, practice-oriented training that addresses the administrative applications of AI, ethical and legal considerations, data protection, professional accountability, and the limitations of AI-supported decisions. Given the perceptual and context-specific nature of the findings, any introduction of AI into special education administration could initially occur through limited pilot applications accompanied by systematic evaluation of accuracy, fairness, legal compliance, and contextual suitability. Decisions affecting students’ services, rights, opportunities, or educational experiences should remain subject to documented human review and final professional responsibility. These recommendations are limited to the context of the present sample and should not be generalized to other educational settings without further empirical evidence.

## Data Availability

The datasets generated and analyzed during the current study are available from the corresponding author upon request. Requests to access these datasets should be directed to Mahmoud Mohamed Eltantawy Department of Special Education, College of Education, Imam Mohammad Ibn Saud Islamic University (IMSIU), Saudi Arabia, mmeltantawy@imamu.edu.sa.
